# Transcriptome analysis of breast cancer in African American women

**DOI:** 10.1186/1471-2105-16-S15-P14

**Published:** 2015-10-23

**Authors:** Shruti S Sakhare, Jamaine Davis, Sammed N Mandape, Siddharth Pratap

**Affiliations:** 1 Bioinformatics Core, Meharry Medical College, Nashville, TN 37208, USA; 2 Dept. of Biochemistry and Cancer Biology Meharry Medical College, Nashville, TN 37208, USA

## Background

Breast cancer is the second most lethal cancer in women. Further, death rates for African American women are the highest for any racial/ethnic group. Hormone receptor status is one of the major prognostic factors and a determinant of treatment options for breast cancer, thus suggesting the importance of molecular level characterization for precision treatments. In this study, we have identified transcriptome level differences correlating to receptor specific molecular subtypes of breast cancer in African American women.

## Materials and methods

Clinical and gene expression data from 18 African American women samples were obtained from The Cancer Genome Atlas (TCGA: http://cancergenome.nih.gov/), yielding transcriptome level analysis between four specific subtypes of breast cancer (Table 1).

**Table 1:** **Sample data classification for receptor specific subtype breast cancer**. (ER = Estrogen receptor, PgR = Progesterone receptor, Her2 = Human EGF receptor 2).

Breast cancer subtype	Receptor status	Receptor status abbreviated
Triple negative	ER^-^PgR^-^Her2^-^	[- - -]
Luminal A	ER^+^PgR^+^ Her2^-^	[+ + -]
Her2 over-expressing	ER^-^PgR^-^Her2^+^	[- - +]
ER positive	ER^+^PgR^-^ Her2^-^	[+ - -]

**Figure 1:**
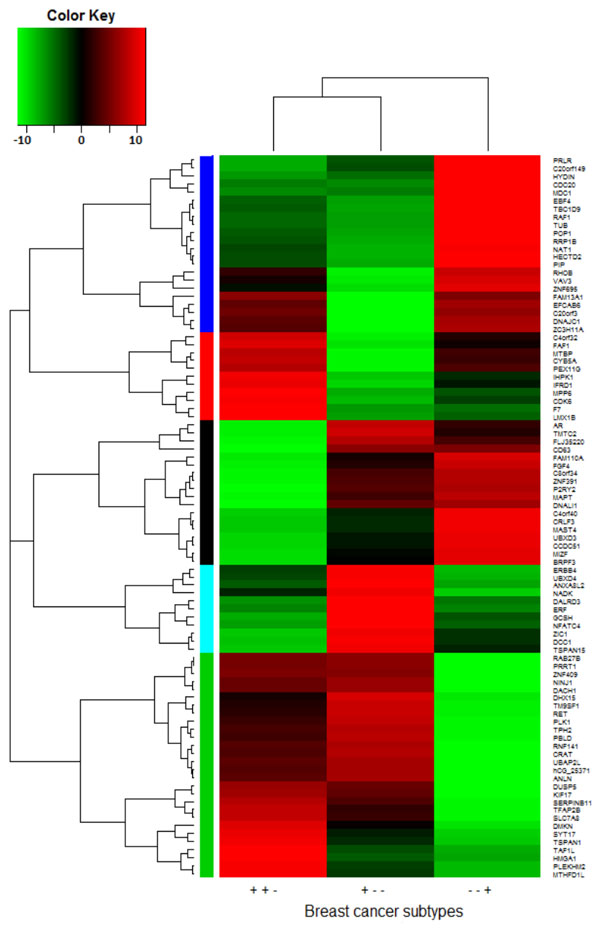
**Hierarchical clustering heat map of significantly altered breast cancer subtype genes in African American women**: Green color indicates down-regulated genes and red color indicates up-regulated genes between differing breast cancer subtypes. + and – of subtype are for ER, PgR and Her2.

**Figure 2:**
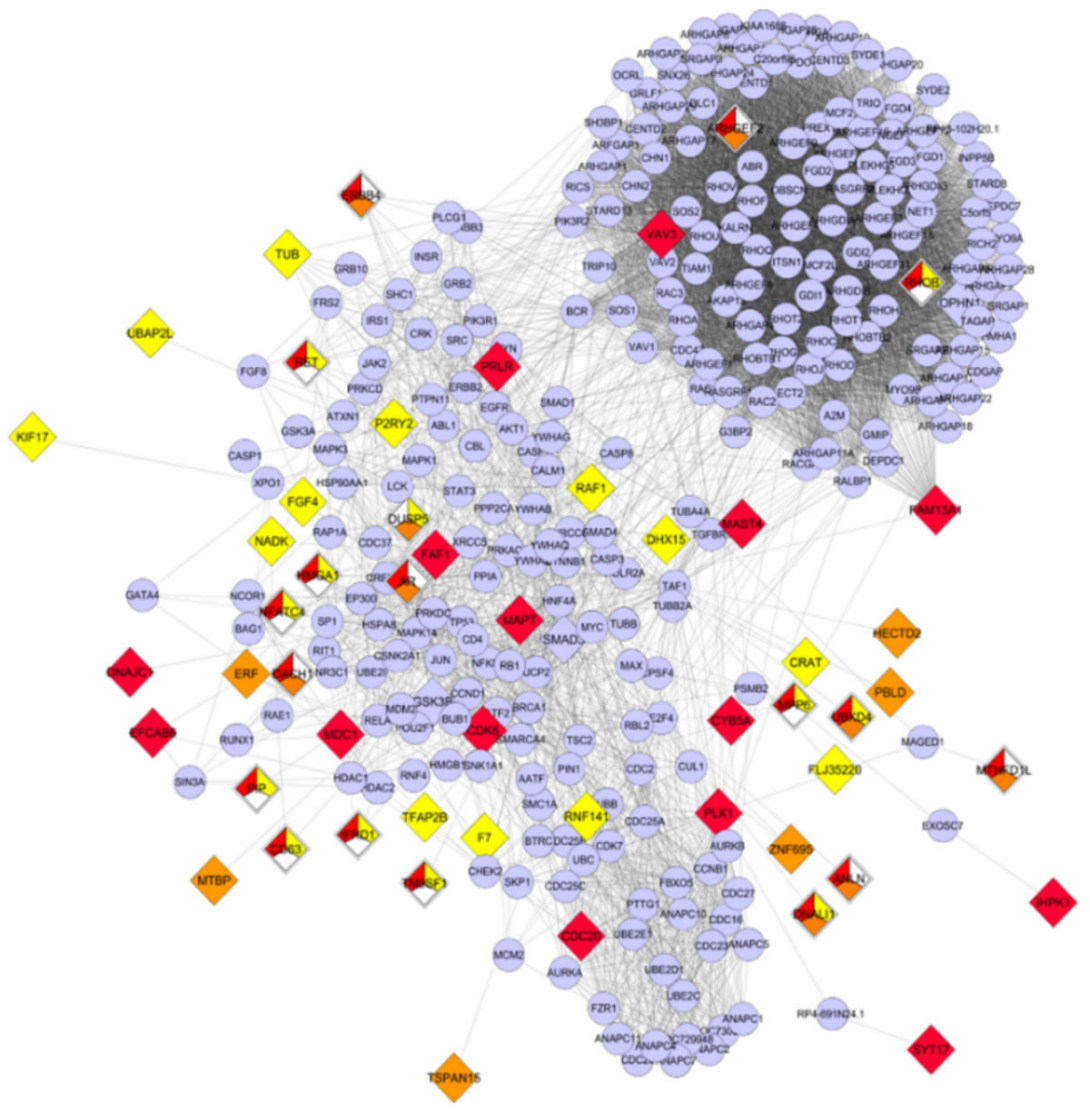
**Interactions network Breast Cancer subtypes**: Diamond nodes are seed nodes of significantly altered gene transcripts varying between Triple Negative, Her2 over-expressing, Luminal A, and ER^+^PgR^-^ Her2^-^ Breast Cancer subtypes in African American Women; circular nodes are 1 degree of biological interactions. Red color indicates genes significant in the group (---,++-), yellow indicates genes significant in the group (--+,---), and orange color indicates genes significant in the group (+--,---).

The samples were analyzed using One-way ANOVA with Welch's correction for unequal sample sizes with type 3 sum of squares. Genes with ANOVA p-value < 0.01 and with relative expression fold change > |2.0| were considered significantly altered; this yielded 90 differentially expressed genes between cancer subtypes in our dataset (Figure 1). Next, we constructed a biological interaction network to impute neighboring genes and proteins using the Michigan Molecular Interactions database (MIMI) and Cytoscape (Figure 2)[1,2].

## Results

The biological interaction network included important DNA repair sub-networks consisting of *BRCA1, SMAD3, SMAD4, EGFR* and *MDC1* genes[3,4]. Specifically, *MDC1* showed altered expression for all subtypes of breast cancer and a significant p-value for the Luminal A subtype compared to the Triple negative subtype. The MDC1 protein has been previously implicated in the DNA damage response[5] and significant changes similar to the ones observed in this study may provide clues in understanding novel ways to treat breast cancer. Clustering of genes among subtypes, based on significance and fold change data, suggest Luminal A and ER positive subtypes are the most similar. Conversely, the least similar subtypes, based on our analysis, was observed between Her2 over-expressing and Triple negative subtypes.

## Conclusions

In conclusion, our results highlight a significant difference in the transcriptome levels of critical DNA repair proteins among the different breast cancer subtypes in African American women. The differentials that were observed stress the importance of molecular level characterizations to understand this disease. Understanding the protein interactions involved in this network will have a major role in predicting best courses of action and aid in precision medicine-based approaches to treating breast cancer.
